# Spider Trait Assembly Patterns and Resilience under Fire-Induced Vegetation Change in South Brazilian Grasslands

**DOI:** 10.1371/journal.pone.0060207

**Published:** 2013-03-28

**Authors:** Luciana R. Podgaiski, Fernando Joner, Sandra Lavorel, Marco Moretti, Sebastien Ibanez, Milton de S. Mendonça, Valério D. Pillar

**Affiliations:** 1 Departamento de Ecologia, Universidade Federal do Rio Grande do Sul, Porto Alegre, Rio Grande do Sul, Brazil; 2 Laboratoire d'Ecologie Alpine, CNRS, Université Joseph Fourier, Grenoble, France; 3 Community Ecology Unite, Swiss Federal Research Institute WSL, Bellinzona, Switzerland; 4 Curso de Agronomia, Universidade Federal da Fronteira Sul, Chapecó, Santa Catarina, Brazil; Michigan State University, United States of America

## Abstract

Disturbances induce changes on habitat proprieties that may filter organism's functional traits thereby shaping the structure and interactions of many trophic levels. We tested if communities of predators with foraging traits dependent on habitat structure respond to environmental change through cascades affecting the functional traits of plants. We monitored the response of spider and plant communities to fire in South Brazilian Grasslands using pairs of burned and unburned plots. Spiders were determined to the family level and described in feeding behavioral and morphological traits measured on each individual. Life form and morphological traits were recorded for plant species. One month after fire the abundance of vegetation hunters and the mean size of the chelicera increased due to the presence of suitable feeding sites in the regrowing vegetation, but irregular web builders decreased due to the absence of microhabitats and dense foliage into which they build their webs. Six months after fire rosette-form plants with broader leaves increased, creating a favourable habitat for orb web builders which became more abundant, while graminoids and tall plants were reduced, resulting in a decrease of proper shelters and microclimate in soil surface to ground hunters which became less abundant. Hence, fire triggered changes in vegetation structure that lead both to trait-convergence and trait-divergence assembly patterns of spiders along gradients of plant biomass and functional diversity. Spider individuals occurring in more functionally diverse plant communities were more diverse in their traits probably because increased possibility of resource exploitation, following the habitat heterogeneity hypothesis. Finally, as an indication of resilience, after twelve months spider communities did not differ from those of unburned plots. Our findings show that functional traits provide a mechanistic understanding of the response of communities to environmental change, especially when more than one trophic level is considered.

## Introduction

Fire is an important disturbance that drives the structure and the interactions of ecological communities in flammable ecosystems [1]. Predicting community resilience and reassembly patterns following disturbances is challenging, but more recently a trait-based functional approach of biodiversity is a promising perspective to reveal the mechanisms behind observed patterns [2], [3]. Beyond who is present (taxonomic identity), and who profits and who vanishes from environmental change (taxonomic turnover), a functional approach may inform: how the organisms are morphologically and functionally structured in the community and how they behave, what they do in ecosystems, and which functional traits are selected or filtered out in face of a disturbance. Such information represents a more generalist [4], comparative [5], [6] and even meaningful [7] view of community diversity which has been gradually incorporated in ecological studies complementing the traditional taxonomic indicators [8], [9]. Furthermore, as the adaptation and function of organisms in their environment are expressed by their traits, functional diversity (FD) may be directly related to ecological niche diversity [7], [10], [11], thereby facilitating the understanding and prediction of community assembly patterns [12], [13].

One theoretical framework on community assembly assumes that local communities are made of organisms assembled according to their physiological, morphological, and/or life-history traits from a regional biodiversity pool [14], [15]. In general terms, on the one hand assembly processes affected by habitat constraints (e.g. environmental filtering) may lead to trait-convergence patterns, while biotic interactions (e.g. competition) may produce trait-divergence patterns [16]. Community trait convergence is generated when in a given site some trait states are favoured instead of others [17]. The result is that organisms under similar environmental conditions will tend to share similar traits compatible with those conditions. For example, reduced soil moisture conditions can select collembolan species that are drought-tolerant, larger-sized (more resistance to desiccation) and with epiedaphic habits [18]. However, trait-divergence occurs when, in order to allow coexistence, the organisms in communities tend to be dissimilar to each other regarding their traits, following limiting similarity (niche differentiation) principles [14], [19]. For instance, ant species present overdispersion in body size at some spatial scales as a way to avoid interspecific competition for similar resources [20], [21]. Trait-convergence and trait-divergence patterns have been often assessed within communities, i.e., considering only alpha functional diversity [22]. Nevertheless, such assembly patterns may be most easily understood if analysed at the metacommunity level (beta functional diversity). Here we adopt this approach by analysing beta functional diversity along ecological gradients [7], [13].

Habitat disturbances remove individuals, or biomass, from a community [23]. Their effects on biodiversity depend on their spatial scale, severity and intensity [24], and also on the ecosystem's resistance and resilience [25]. Fire is one of the major disturbances driving vegetation physiognomy and structure [1], and especially in fire-prone ecosystems it plays a key role in the selection of adaptive functional traits in plants [26]. As fire opens the vegetation, it partially resets community assembly processes reducing competitive dominant plant species and may thus increase plant species richness and FD by allowing less competitive and functionally diverse species to establish [27], [28], [29]. Responses of terrestrial arthropods and other animal groups to fire tend to be idiosyncratic, and usually depend on their colonization capacity and the suitability of the habitat as a result of vegetation regrowth and soil recovery [30]. Studies addressing such mechanisms of reassembly in animal communities considering a functional approach are limited (e.g. [31]), and therefore a complete understanding of the role that the post-burn habitat structure plays in animal assembly is required.

Here we examine how disturbance by fire affects the assembly of spider communities from a functional perspective. Spiders are abundant and diverse generalist predators in most terrestrial ecosystems [32], playing a potential role in biological control and insect suppression [33]. In contrast to any other predator, spiders display a wide range of complementary foraging strategies reflecting their essential relationship with the vegetation and habitat structure [34], [35]. We conducted a replicated burning experiment in fire-prone natural grasslands of South Brazil where besides the description of spider communities we collected information on the functional traits of the plant communities as a surrogate of habitat structure characterization. We aim at (1) assessing the resilience of spider communities to fire (e.g. the time needed by spider communities to recover after fire); (2) detecting relationships between spider and vegetation communities from a functional perspective in burned and unburned sites, and (3) exploring spider trait-convergence and trait-divergence on gradients of plant biomass and plant functional diversity generated by fire. For this we used trait data collected on spider individuals, which enabled us to consider the entire trait variability between individuals [36], including variability related to phenology and sex, both within and between experimental treatments. We showed that fire induced changes on vegetation traits led to spider trait community patterns indicative of environmental filtering and limiting-similarity processes, which lasted less than one year after the disturbance.

## Materials and Methods

### Study area

We conducted the experiment in a natural grassland site at the Agronomic Experimental Station of UFRGS, Eldorado do Sul municipality, Rio Grande do Sul, Brazil (30°06′58″S; 51°41′05″W). The grasslands in South Brazil, regionally known as *Campos*, are located in a transitional zone between tropical and temperate climates (Cfa type according Peel et al. [37]). The mean temperature ranges from 9°C in winter to 25°C in summer, and the annual precipitation is about 1440 mm normally well distributed in the year [38]. The vegetation structure is dominated mainly by grass species with co-occurring herbs, shrub and treelet species.

Campos represent ancient ecosystems from colder and dryer, or warmer and more seasonal climates that prevailed until the mid-Holocene [39]. As the climate became moist and milder it favored forest expansion over the grasslands. Nowadays, disturbance by grazing and fire controls woody plants encroachment and has maintained the native grassland physiognomy and diversity of these ecosystems in South Brazil [40]. Despite burning prohibition by Brazilian environmental legislation, farmers lit fire as a management tool to eliminate dead grass biomass and increase forage quality [41]. In association with grazing, which creates small-scale heterogeneity of grazed and ungrazed patches [42], fire is usually of low intensity and spreads rapidly and heterogeneously according to available flammable biomass and wind conditions, creating a mosaic of burned and unburned patches [41]. Further, most of the plant species in these grassland ecosystems are perennial and vegetation recovery after fire is usually fast due to resprouting [43], [44].

### Experimental design

Our experiment comprised 14 paired plots of 10×10 m (seven blocks), disposed in grassland areas with a gentle slope. Blocks of paired plots were separated by at least 50 m, and the plots from the same block were six meters apart. One plot per block was randomly burned at late spring of 2009, and the other plot served as a control. The prescribed burnings were authorized by the Environmental Secretariat of Rio Grande do Sul state (SEMA, Brazil), and controlled with firebreaks surrounding the plots.

We opted to use small scale burned plots (10×10 m) that mimic the fire mosaic as it occurs in Campos instead of larger ones because (1) homogeneity was required inside each plot and between plots of the same block, and (2) in small plots we can reset assembly processes avoiding the effects of distance for the colonization of the organisms; e.g. the smaller the plots and larger the surrounding matrix, the lower the requirements on arthropod dispersal ability and the more the results relate to their habitat preferences [30].

### Spider sampling

We sampled the spider community in all the plots before the application of the treatments, one month after fire, between six to seven months after fire (early winter), and twelve months after fire. We collected the spiders from vegetation with sweep nets and from the soil surface with five equalized pitfall traps per plot. The sweep net was 50 cm large (0.1 m^2^); we swept the vegetation in four transects in each plot in one morning and one afternoon at each sampling date. Pitfall traps consisted in plastic pots (9 cm diameter) filled with 200 ml of alcohol 70% and some drops of detergent; they remained opened for four days in the field. As pitfall traps measure the activity density of the wandering organisms on the soil surface, we are aware of a bias in the effects of habitat openness in their trapability (probability of individual capture; [45]); i.e. trapability could increase in recently burned plots because there are fewer constraints to locomotion. We take this into account for interpretation of our results. The sampling was authorized and registered at ICMBio/SISBIO under the process number: 20579-1.

In the laboratory, we counted all the spiders and sorted them to adults or juveniles. As 82% of all individuals sampled were juveniles, which are difficult and fundamentally ambiguous to identify to species level [46], especially in mega diverse countries, we based our taxonomical approach at family level. In Brazil spiders are very rich in families which are relatively easy and hence fast to identify, even considering immature individuals; e.g. the study region (Rio Grande do Sul state) hosts 51 spider families [47]. Significant correlation has been confirmed by previous studies between family and species richness [48], and response to disturbances, including fire [49].

### Spider traits

We assessed the functional response of spiders to fire by using behavioral and morphological traits documented for each spider individual (Table 1). We classified the spiders according to their behavior of building or not building a prey-capture web (web builders or hunters), and in a second step we sorted the web builders by their type of web: (a) orb-web, or (b) irregular-web (others than orb-web; e.g. sheet and spatial-webs), and the hunters by their living strata: (c) ground or (d) vegetation hunters. It is assumed that these four spider foraging trait strategies present different responses to environmental factors (e.g. habitat structure) and also undergo different effects on ecosystem processes (e.g. differential predation) [50], [51], and therefore are likely relevant to examine convergence and divergence patterns. The foraging strategies were considered as binary traits (Table 1), and were based primary on family affiliation [34], [35] (Table S1), and in the organism living strata (ground and vegetation hunters). For example, Miturgidae and Salticidae sampled with pitfall traps were classified as ground hunters, and those collected by sweeping net as vegetation hunters.

**Table 1:** Description of spider and plant traits used in the study.

Group	Trait category	Trait (abrev.)	Category	Definition
Spiders	Morphological	body size (body)	quantitative	(cephalothorax length*width)+(abdomen length*width)
		leg size (leg)	quantitative	(average of anterior and posterior femur)/cephalothorax length
		eye size (eye)	quantitative	Larger frontal eye width/cephalothorax width
		chelicerae size (chel)	quantitative	(chelicerae length*width)/(cephalothorax length*width)
	Feeding behavior	ground hunters (gh)	binary	ground hunters = 1; other = 0
		vegetation hunters (vh)	binary	vegetation hunters = 1; other = 0
		orb web (ow)	binary	orb web = 1; other = 0
		irregular web (iw)	binary	irregular web = 1; other = 0
Plants	Morphological	plant height (pl_he)	quantitative	plant height
		leaf area (le_ar)	quantitative	leaf area
		leaf lengh (le_le)	quantitative	leaf length
		leaf width (le_w)	quantitative	leaf width
	Life-form	graminoids (gram)	binary	graminoids = 1; other = 0
		forbs (for)	binary	forbs = 1; other = 0
		rosettes (ros)	binary	rosettes = 1; other = 0

Spiders were also described by morphological traits (body, eye, leg and chelicerae size) also assumed to be related to their adaptation and function in the environment. For example, body size is correlated with many life history mechanisms as resource use, starvation, desiccation resistance, and other physiological processes [52], [53]. Eyes are connected with the collection of visual information about microhabitat features and substrate, hunting and spatial orientation [54], [55]; leg size could be related to efficiency in locomotion, dispersal and web construction [32], [56]; and finally chelicerae size could refer to prey size. Morphological measurements were done on each spider individual with micrometer under stereomicroscope. The measurements of each structure (leg, eye, and chelicerae) were weighted by a relative body size measurement (Table 1); *e.g.* chelicerae area was divided by cephalothorax area. See Table 1 for a complete description of the measurements.

### Vegetation sampling

The experimental plots were sampled before fire to test initial vegetation homogeneity between paired plots. We sampled the vegetation again in all experimental plots approximately nine months after fire (late winter/early spring) to examine spider community responses to fire through changes in plant community functional structure. We used this last data set for comparison with the post-fire winter spider data. In both occasions we calculated the mean cover (%) of each plant species and bare soil that were visually estimated in five 1 m^2^ quadrats randomly distributed in each plot at each date. Plant aboveground biomass was assessed in each quadrat by cutting and weighting fresh biomass. An aliquot from total biomass was oven-dried (60°C for 72 h) and weighed for total dried biomass estimation. The sampling quadrats within plots were randomly located at the beginning of the experiment and were non-overlapping. For the data analysis we used the average species composition and biomass of each experimental plot and period.

Plant species were described by traits that could affect structural features of the habitat offered to spiders, that is, plant life-form (graminoid, forb or rosette), and other morphological traits (plant height, leaf area, leaf width and length; Table 1). The traits were recorded for the most dominant and most frequent species, that is, those with a minimum cover of 10% in at least one of the quadrats and those occurring in at least 30 among the 140 evaluated quadrats. In this way, traits for 52 species, corresponding to 46% of the total species pool (114 species), comprising up to 88% of total plant cover were recorded. For plant morphological traits we used the average of measurements on five individuals from each species collected from the whole study area. Information on life-form was collected from the literature and considered as binary traits (Table 1).

### Data Analysis

In data analysis, we (1) tested for effects of fire on the spider and plant communities described by taxon identities and by their traits, (2) examined the relationships between spider traits and vegetation traits, and (3) identified spider assembly patterns related to vegetation gradients.

#### Data matrices

For the analysis, we organized the spider and vegetation data collected at each sampling period in the following matrices: matrix **B**_S_ of spider individuals by traits, binary matrix **W**_S_ of experimental plots (in rows) described by the presence of the spider individuals, which was standardized to unit total within experimental plot and period, and matrix **F**_S_ with the same plots described by the abundance of individuals classified in spider families. Similarly, the vegetation data was arranged in matrix **B**_P_ of plant species by traits, matrix **W**_P_ of experimental plots by the cover of plant species (also standardized to unit total within experimental plot and period), and matrices **E**_PB_ and **E**_FD_ (actually vectors) respectively with aboveground plant biomass and calculated plant functional diversity (see below) in the experimental plots. These matrices are illustrated in Figure S1.

#### Community mean traits and diversity

For each plot and sampling period we calculated community weighted mean traits (CWM) for spiders and for plants. CWM trait values represent the mean of each trait weighted by the relative abundance *p_i_* of the *i*-th spider individual or plant species presenting each trait value *x_i_* [57], [58]. Such metric informs on dominant traits in the community, which is related to the “mass ratio hypothesis” [59]. CWM trait values were computed by matrix multiplication **T** = **WB** [13], where **W** and **B** were defined according to spiders or plants and sampling period. Matrix **T**_S_ and **T**_P_ will contain, respectively, spider and plants CWM trait values (see Figure S1).

For each plot and sampling period we also calculated functional diversity (FD) for spiders and for plants using Rao's quadratic entropy [60], [61], which informs on the extent of trait dissimilarity among taxa in the community, and it is linked to the “limiting similarity” [14], [16] and “niche complementarity hypothesis” [62]. In this way FD is calculated as the sum of the dissimilarities *d_ij_* based on traits weighted by the product of the relative abundances *p_i_* and *p_j_*. of the *i*-th and *j*-th spider individuals or plant species:
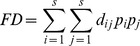



This sum is carried over all pairs of spider individuals or plant species found in the experimental plot at a given sampling period. For the dissimilarities *d_ij_*, which should be defined in the range 0 to 1, we used the Gower's similarity index [63]. For spiders we considered all functional traits together (FD_S__all traits), and, because of their different natures, we also calculated FD for spider feeding behavior (FD_S__behav) and morphological traits (FD_S__morph), separately. For plants, we calculated FD indices considering all plant traits together (FD_P__all traits) and also separately for life-form (FD_P__life-form) and morphological traits (FD_P__morph). Additionally, we computed the Simpson index of spider family diversity (based on matrix **F**_S_).

#### Fire effects and spider community resilience

We assessed the effects of the experimental treatments on vegetation variables before fire and nine months after fire using analysis of variance based on randomization testing (1000 permutations). We considered plant species composition (matrix **W**_P_) in one test, and CWM traits (matrix **T**_P_ for seven traits) in another, and in these cases the analysis was multivariate. We also tested for treatment effects on total plant species richness, aboveground biomass (log transformed), FD and each CWM traits, in which case the analyses were univariate.

We applied the same randomization method to test for effects of the treatments on spider community variables at each sampling period (four periods). We considered spider family abundances (matrix **F**_S_), and CWM traits (matrix **T**_S_ for eight traits). Family abundances were log transformed (x+1) to reduce the effects of very abundant families. Using the same method we also compared burned and unburned plots in terms of individual variables: (1) spider abundance, (2) Simpson index of spider family diversity, (3) Bray-Curtis similarity coefficients of spider families in all pairs of plots, (4) FD (all traits, behav and morph), and (5) CWM traits. The comparison of the results of the tests across sampling periods allowed assessing resilience of the spider communities to fire.

#### Relations between spider traits and vegetation traits

To explore the association between functional traits of spiders and plants we used co-inertia analysis [64]. We tested the co-variation between matrix **T**_S_ of spider CWM traits and matrix **T**_P_ of plant CWM traits. **T**_S_ in this case was based on the spider individuals collected between six to seven months after fire, and **T**_P_ on the plant species composition recorded nine months after fire, in both burned and control plots. Firstly we performed PCA of the two matrices and selected for each of them the principal axis to reduce their dimensionality. Secondly, the concordance between the two data sets was maximized by the rotation of the multidimensional space, generating new axes [65]. Finally, the significance of these associations was tested by permutation. To interpret the relationships between specific traits of spiders and plants in the treatments we also plotted their CWM traits in two separated PCAs. For interpretation, we only considered those traits with statistically significant responses to burning (see previously described ANOVA).

#### Spider assembly patterns

We analysed spider assembly patterns following the method described by Pillar et al. [13], which distinguishes trait-convergence (TCAP) and trait-divergence assembly patterns (TDAP) along an ecological gradient. Here we sought TCAP and TDAP in spider communities using plant biomass or plant FD in burned and unburned plots as ecological gradients possibly driving the assembly process. Since not every measured trait may be related to the environmental gradient considered, we searched for optimal trait subsets maximizing the expression of such patterns. Then, the selected traits were used for testing and interpreting the patterns.

TCAP is evaluated by a Mantel type correlation of dissimilarity matrices based on **T** and **E**, i.e. ρ(**TE**) = ρ(D**_T_**;D**_E_**), which measures the congruence between variation of spider CWM traits (**T**_S_) and the variation of plant biomass (vector **E**_PB_) or plant functional diversity (vector **E**_FD_ for FD_P__all traits). The correlation ρ(**TE**) approaches 1 as more communities that are similar due to spider traits are also similar regarding plant biomass or plant functional diversity. In this case, changes in the traits are linked to the gradient and therefore organisms within communities nearby on the gradient will tend be more similar to each other than organisms in communities far apart on the gradient [13]. TCAP was tested against a null model, which was based on the permutation between the row vectors (spider individuals) of matrix **B**_S_, generating a permuted matrix **B**_Srnd_. At each permutation, a new matrix T_rnd_ = **WB**_Srnd_ and the corresponding ρ(**T**_rnd_ **E**) are recomputed. After many permutations (at least 1000), the probability of finding under the null model a ρ(**T**_rnd_ **E**)≥ρ(**TE**) is found. See Pillar et al. [13] for further details.

TDAP evaluation at the metacommunity level requires, as an intermediate step, computing matrix correlation ρ(**XE**) [13]. For this, we obtained matrix **U** with degrees of belonging of spider individuals to fuzzy sets [66] based on the individuals' trait similarities computed from matrix **B**_S_. With this approach, we consider each organism as defining a fuzzy set to which itself and every other organism may belong with a certain degree of belonging ranging in the interval [0, 1] [67]. The idea behind the definition of fuzzy sets in this context is that organisms that are similar by their traits are functionally equivalent and could replace each other in the communities. By matrix multiplication, **X** = **U'W** is defined containing the plots' spider composition that is fuzzy-weighted by the spider individual similarities. Matrix **X** is actually indicating the probabilities for every individual being present in the plot given its similarity to the individuals that were actually found in the plot. Since matrix **X** carries the whole information of the organisms' traits, that was transferred from the organism level to the metacommunity level, the matrix contains both convergence and divergence patterns [13]. Therefore, the Mantel partial correlation ρ(**XE**.**T**) will express only spider TDAP strictly related to plant biomass or plant FD. ρ(**XE.T**) was tested against a null model based on the permutation between the rows of **U**, analogously to the testing of ρ(**TE**). See Pillar et al. [13] for further details.

Optimal trait subsets maximizing the values of ρ(**TE**) and ρ(**XE.T**) were obtained through an algorithm that considered all trait combinations starting with one trait, up to the complete set of traits [68], [13]. After this procedure we evaluated the significance of ρ(**TE**) and ρ(**XE.T**) obtained with the optimal trait subsets related to each gradient.

After identifying significant sets of optimal traits for TCAP, we plotted CWM of each trait of the optimal subset and the ecological gradient. Also, matrix **X** defined by the optimal trait subset maximizing ρ(**XE.T**) was analyzed by Principal Coordinates Analysis (PCoA). PCoA was performed with Euclidean distances between sampling units, bi-plotting traits (CWM) and the related environmental variable (**E**).

All analyses but trait-assembly pattern analysis were performed with R 2.15.1 [69]. For trait-assembly pattern analysis in ecological gradients we used the software SYNCSA (available in http://ecoqua.ecologia.ufrgs.br), but also available in the package SYNCSA for R.

## Results

### Plant and habitat structure

Before fire we identified a total of 114 plant species in the study site, and an average of 20 plant species per quadrat of 1 m^2^. The average aboveground biomass was 817 g per m^2^. Analysis of variance indicated that before fire the experimental plots from the same block were not different from each other concerning plant parameters (Table 2 and Table S2). The fire consumed essentially all leaf-litter material and green plant biomass in the plots, leaving only some partially unburned *Eryngium horridum* (Apiaceae) individuals. Plant biomass decreased in burned plots (P = 0.005), and plant total richness increased (P = 0.015). Treatments did not differ in both dominant plant species composition (P = 0.237), overall plant trait variation (FD_P__all traits) and plant morphological variation (FD_P__morph), while plant life-form variation (FD_P__life-form) increased in burned sites (Table S2). Dominant traits in the community (CWM) also differed between treatments, with a significantly higher proportion of rosettes and plants with broader leaves in burned plots, as opposed to more graminoids and taller plants (Table S2) in unburned control plots. A complete description of habitat structure after fire at the sampling dates is given in Table 2.

**Table 2:** Vegetation variables in burned and unburned experimental plots at different sampling periods.

Sampling periods	Burned compared to control plots
Before fire	Similar plant composition, richness, FD and CWM traits^a^
**Prescribed Fire** (late Spring 2009)	
Short-term(1 month after fire)	Sparse vegetation regrowth, flowering induction, decreased litter and increased bare soil cover^b^
Intermediate-term(6 to 9 months after fire)	Similar plant species composition; increased plant species richness, FD_life-form and proportions of rosettes and broader leaves; decreased plant biomass, proportions of graminoids and tall plants^a^; decreased amount of litter on the ground^b^
Long-term(upper to 12 months after fire)	Similar vegetational parameters^b^

a showed experimentally based on our measurements.

b qualitative observations, and according to Fidelis et al. [44].

### Spider data description

During our study we collected a total of 23 spider families and 1755 individuals (Table S1). Among these, 15 families and 80% of the individuals were sampled directly from vegetation, and 22 families and 20% of the individuals from the soil surface. The families with more than 5% of the total abundance of individuals were Araneidae (22.8%), Thomisidae (18.4%), Salticidae (15.4%), Oxyopidae (13.5%) and Lycosidae (7.5%). Vegetation hunters accumulated 54% of the total individuals collected, followed by orb web builders (24%), ground hunters (11%), and irregular web builders (11%; Table S1).

### Spider Resilience

Before fire paired plots did not differ from each other concerning all spider biodiversity indices evaluated (Table 3). After fire, we also did not find any significant difference between treatments at any sampling date concerning spider abundance (Figure S2A) and Simpson diversity (Figure S2B). Bray-Curtis similarity in terms of family composition between burned and unburned communities remained similar during all sampling periods (approx. 68% of similarity, Figure S2C). The spider community composition described by families (P<0.001) and by CWM traits (P<0.001) presented strong seasonal dynamics, with spiders being more dissimilar between sampling dates than between treatments. However, trait composition seemed to be dissimilar between treatments one month after fire (P = 0.019).

**Table 3:** Summary of the results of spider functional resilience to fire.

Functional measures of spider community		p-values (fire effect)			
		Before fire	1 month a.f.	6–7 months a.f.	12 months a.f.
FD	All traits	0.961	0.667	0.414	0.441
	Morphological	0.823	0.321	0.330	0.387
	Feeding behavior	0.092	**0.038 (−)**	0.745	0.961
CWM	All traits	0.634	**0.019**	0.158	0.994
	Body size	0.280	1.000	0.667	0.706
	Leg	0.745	0.745	0.521	0.863
	Chelicerae	0.804	**0.035 (+)**	0.745	0.194
	Eye	0.594	0.342	0.477	0.686
	Orb web	0.941	0.120	**0.041 (+)**	0.863
	Irregular web	0.122	**0.019 (−)**	0.686	0.623
	Ground hunter	0.745	0.706	**0.003 (−)**	0.823
	Vegetation hunter	0.311	**0.027 (+)**	0.391	0.961

Probability values from analysis of variance in blocks with permutation test obtained for functional measures of spider community between control and burned plots at different sampling dates before and after fire (a.f.). For CWM_all traits the analysis was multivariate. In case of significant differences, positive or negative effects of fire are showed in brackets.

Regarding functional aspects, FD of feeding behavior (FD_S__behav) decreased in burned plots one-month after fire, while there were no differences in the variation of both the overall traits (FD_S__all traits) and morphological traits (FD_S__morph). The analysis of spider CWM traits revealed that one month after fire there was a positive effect of fire on chelicerae size and vegetation hunters, and a negative effect on irregular web builders. At six to seven months after fire we found negative effects of fire on ground hunters and positive effects on orb web builders (Table 3). At 12 months after fire the functional traits of the spiders did not differ between treatments.

### Relationships between spider traits and plant traits in burned and control plots

**Figure 1:**
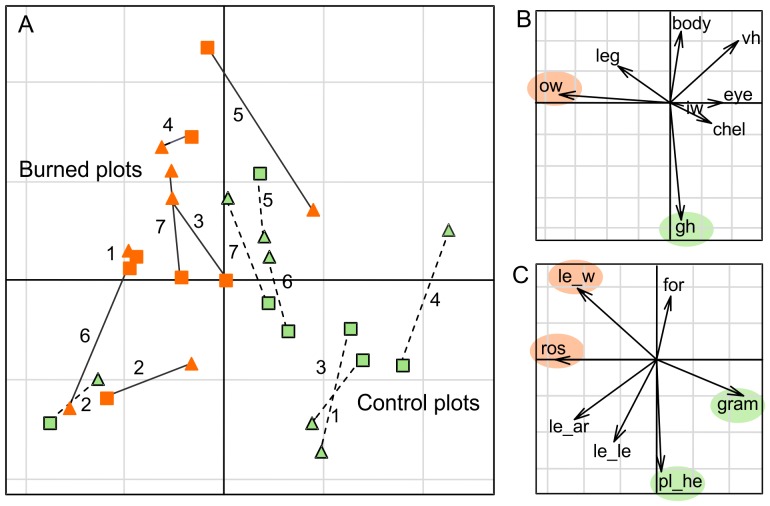
Relationships between spider traits and vegetation traits in burned and control plots. Co-inertia analysis results: (A) ordination of control (light-green symbols) and burned plots (dark-orange symbols) based on spider (triangle) and plant traits (square); PCA of (B) spider and (C) plant traits. Short arrows indicate that the plant and animal traits occupy similar positions in the ordination space. Numbers (1 to 7) indicate the blocks. Highlighted traits (B, C) mean significant association with burned or control plots.

Co-inertia analysis of the simplified dimensional data of spider traits×plots (PCA, five eigenvectors, 92.91%) and plant traits×plots (PCA, four eigenvectors, 96.83%) showed marginally positive association between these two datasets (RV = 0.40; p = 0.068; Figure 1A). The first and second axes of the co-inertia biplot clearly reflected the fire treatment, and represented 68.3% and 22.9% of the co-structure respectively, with unburned control plots in the bottom right corner of the ordination and burned plots in the top left corner The blocked design of the fire experiment was reflected by closeness of the paired plots (burned and control) in the ordination space.

Figures 1B and 1C show the contribution of the functional traits of the two trophic levels to the canonical space. Considering only the statistically significant traits of spiders and plants (Table 3 and Table S2), in burned plots orb web builders correlated positively with rosettes and plants with broader leaves. On the other hand, ground hunter spiders were associated with taller plants and graminoids in unburned plots.

### Spider assembly patterns in plant ecological gradients

**Figure 2:**
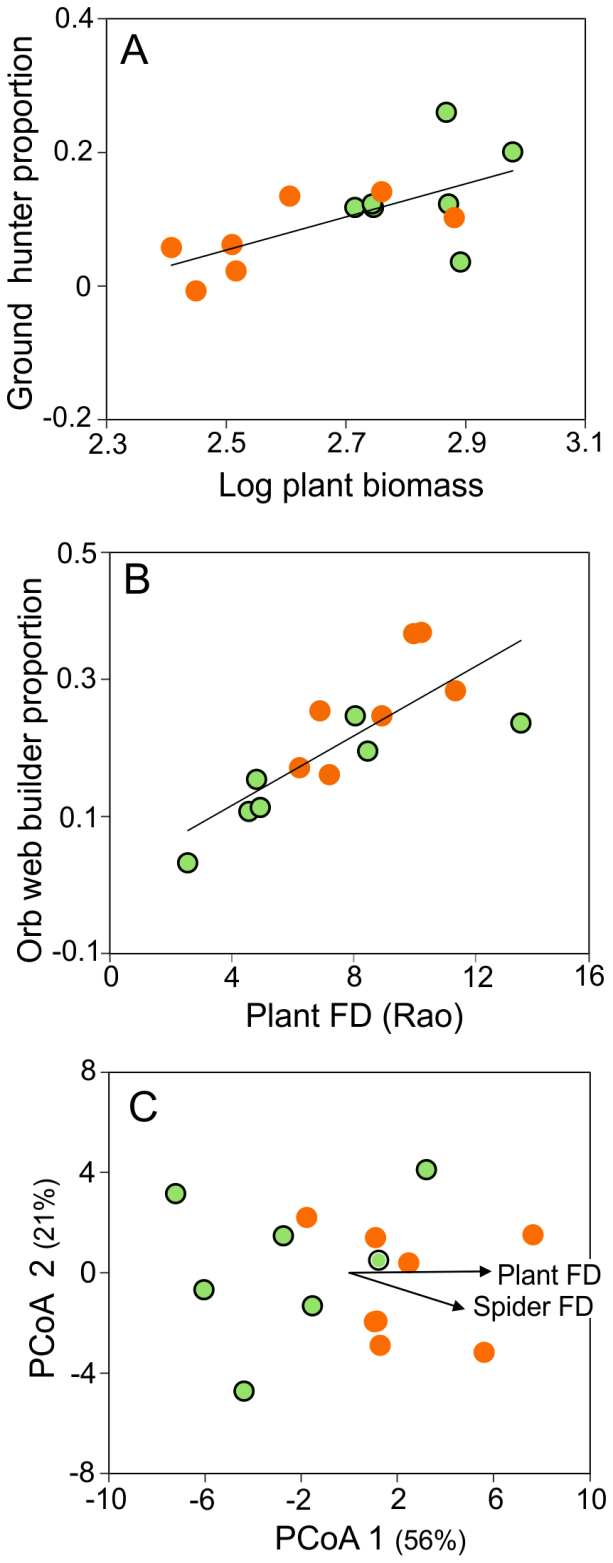
Spider assembly patterns in ecological gradients. Trait convergence assembly pattern (TCAP) under gradients of plant biomass (A) and plant FD (B). Trait divergence assembly pattern (TDAP) in gradient of plant FD (C). The TDAP plot is composed of an ordination diagram generated on Euclidian distances computed on the composition of spiders communities after fuzzy weighting by the traits which maximized the expression of TDAP (body, chel, eye, iw, ow) related to plant FD. Green symbols mean control plots and orange symbols mean burned plots.

We evaluated trait assembly patterns in spider communities related to two ecological gradients: plant biomass and plant FD. Spider trait-convergence assembly pattern (TCAP) related to plant biomass was maximized by the ground hunter attribute (Table 4). The proportion of ground hunters and plant biomass were positively associated, and lower in burned plots (Figure 2A). For example, with a decrease in biomass from 934 to 263 g/m^2^, the proportion of ground hunters decreased from 0.18 to 0.05.

**Table 4:** Summary of the results of spider assembly patterns in ecological gradients.

Gradient	TCAP				TDAP			
	Biomass		FD plants		Biomass		FD plants	
Optimal trait subset	gh		ow, eye, chel		leg, chel, eye, body		body, chel, eye, iw, ow	
ρ(**TE**)	**0.335**	**(0.015)**	**0.553**	**(0.001)**	0.047	(0.337)	0.105	(0.268)
ρ(**XE.T**)	−0.147	(0.919)	−0.140	(0.49)	0.152	(0.157)	**0.534**	**(0.002)**

Spider trait subsets maximizing, at the community level, the expression of trait-convergence assembly patterns (TCAP) and trait-divergence assembly patterns (TDAP) related to plant biomass and plant FD ecological gradients. See Table 1 for descriptions of trait abbreviations.

Along the plant FD gradient, spider TCAP was significantly maximized by orb web builder, chelicerae and eye size attributes (Table 4). By plotting these traits against the gradient of plant FD we observed that orb web builders had a primary importance for the expression of TCAP, and chelicerae and eye size appeared only to participate explaining the residuals of the main correlation. The proportions of orb web building spiders increased with plant FD (Figure 2B); i.e. with an increase from 5 to 12 in plant FD (Rao Index) the orb web builder proportion increased from 0.14 to 0.32.

No significant spider trait-divergence assembly pattern (TDAP) was found regarding plant biomass gradient (Table 4). However, we found a strong divergence pattern related to plant FD maximized by chelicerae, eye and body size, orb and irregular web builders. Spider FD of these traits was positively correlated to plant FD, which tended to increase in burned plots (PCoA; Figure 2C).

## Discussion

Our findings indicate that spider communities can recover very fast to plot-scale fire in grasslands of South Brazil. Although we visually detected the arthropods disappearance after fire, either because they were consumed by fire or escaped, we observed that the spider communities of burned sites were similar in number of individuals, family diversity and composition to those in unburned sites from the first month after fire. As initially predicted, our small scale burned sites appeared to not impose major constraints for spider colonization allowing both cursorial and aerial immigration. While cursorial dispersal represents directional spider short distance travels [70] which probably took place through the soil surface and the resprouting foliage directly from adjacent unburned areas, aerial ballooning is a passive mechanism of distant and random dispersal through air currents [33], [71]. These colonization processes seem to have happened faster than our first sampling (one month after fire) could record, and hence we recommend that future researches perform samplings in an earlier period. We therefore believe that our results indicate spider resilience through fast recolonization rather than spider resistance to fire. The spider families represented at the site had good dispersal abilities and our results also indicated that the postfire ecosystem was capable of maintain such diversity. Species with restricted dispersal abilities and habitat specialists were probably disfavored in this initial colonization process [71], [72]. Because recently burned habitats would offer less niche diversity than unburned habitats, a decrease in arthropod species diversity overall is expected [30], [73], but our approach at family level was not suitable to detect such responses.

Functional diversity is a dimension of biodiversity which takes into account the traits of organisms related to their adaptation in their environments [10], and thus can inform on their biological mechanism of response. Our functional approach revealed short and intermediate-term responses of spiders to fire, with a closer similarity between burned and unburned areas only reached after about one year, probably reflecting the time frame for recovery of the vegetation [29]. Our results therefore indicate spider communities are functionally resilient to fire disturbance in these grassland ecosystems so that they recover within one year after fire.

In the first month after fire we observed a reduction in the relative abundance of irregular web builders and an increase in that of vegetation hunters, which resulted in a decrease in FD of spider feeding behavior. On the one hand, irregular web-builders could have been limited in recently burned sites because of shortage in appropriate physical structures for attachments of their webs. This functional group incorporates spiders that build especially sheet and spatial webs and, in contrast to orb weavers which are more flexible and can spin webs across wider spaces [74], [75], they may require relatively small distances between web supports [51], having a close relationship with microhabitats in the litter layer [76], [77] and plants with dense foliage [78]. Recently burned sites had sparse vegetation and a huge simplification of the ground surface, with reduced litter and moisture [44] which seemed to compose an unsuitable habitat for these spiders.

On the other hand, vegetation hunters were favored in this environment at this early period probably by the direct vegetation regrowth and the summer-induction of flowering of many inter-tussock dicot species that established following release from dominance by grasses and suppression of the thick litter layer [29], [40], [79]. Freshly green biomass and flowers represent optimal foraging patches for wandering spiders by attracting potential preys such as flower visitors, pollinators [80], [81] and herbivore insects which feed preferentially on young high-quality leaves vs. mature ones [82], [83], [84]. Interestingly, also at this time the spiders found in burned plots had larger chelicerae size, but not body size, than in unburned plots which may be explained by an increment in prey size in the post-burned habitat.

**Figure 3:**
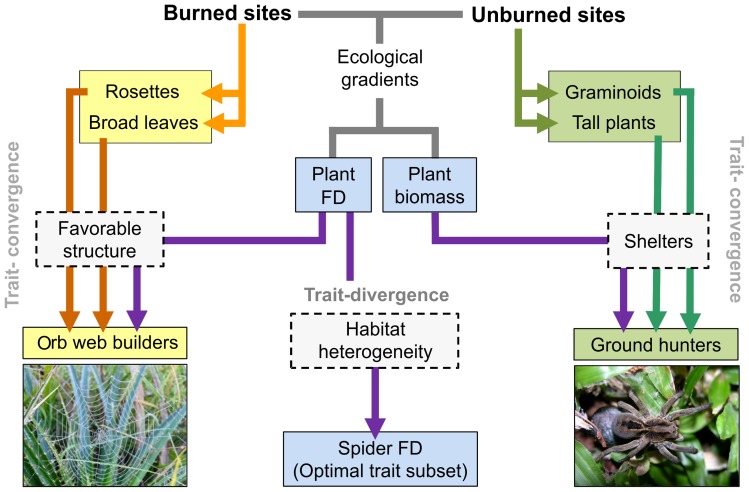
Conceptual map of the main findings of the study for an intermediated time after fire. Unburned plots were most characterized by graminoid-form and taller plants leading to increased proportions of ground hunter spiders. Plant biomass gradient also acted as an important environmental filter for this kind of spiders probably by maintaining proper conditions of shelters and microclimate in soil surface. On the other hand, burned plots presented increased proportions of rosette-form plants with broader leaves which favored spiders building orb webs; this hunting strategy also converged positively in the plant FD gradient influenced by suitable vegetation structure to attach their webs. Additionally, spider individuals occurring in more functionally diverse plant communities were more functionally diverse concerning their traits (body, chelicerae and eye size, and web type building) because functionally diverse plant communities provide more ecological niches and increased possibility of resource exploitation, following the habitat heterogeneity hypothesis. Photo in the left: Araneidae web in *Eryngium horridum* (Apiaceae) by Denise Dell'Aglio; Photo in the right: *Lycosa erythrognatha* by Estevam Cruz.

At an intermediate time after fire, beyond a reduced plant biomass and litter layer we confirmed the generally accepted pattern of increasing plant species richness in burned sites. Moreover, this pattern was associated with an increase in FD of plant life forms. It is already known that the dominance of C4 tussock grasses tends to reduced plant diversity in undisturbed grasslands, and that fire, or other grassland management actions like mowing, allow the coexistence of more species from different life forms by reducing the superior competitive species (e.g. [28], [29], [85]). We experimentally showed that unburned areas had greater proportions of graminoid and taller plant species than burned ones, which presented enhanced proportions of rosettes. Rosettes in this study were represented mostly by *Eryngium horridum* (Apiaceae), a very common species in grasslands in southern Brazil [86] that shows a great capacity of regeneration by resprouting after plant damage [87]. This differential plant trait composition between burned and unburned sites as well as the gradients of plant biomass and plant FD along the treatments acted as environmental filters leading to convergence patterns of spider feeding behavior (Fig. 3). Below we detail the mechanisms through which such convergence may operate.

The proportion of ground hunters increased under dense tussocks and tall plants, increased plant biomass, and litter layer (Fig. 3). This probably reflected protection against light and/or visual predators, buffered microclimate, as well as increased surface for moving around, hunting and sheltering, which has a major importance for reduction of mortality risk [88]. Ground hunters are particularly known for selecting their microhabitats on the soil surface based on suitable microclimate, reduced predation risk and availability of prey [89], [90], [91], and usually increase when the biomass and litter layer are enhanced [50], [92]. According to these premises, we would have expected a decrease in these spiders at the simplified recently burned sites, but there were no significant differences at that time. Two possible explanations are that 1) the trapability by pitfall traps may be increased because burned plots offer less constraints to animal locomotion and activity in the soil surface [45], compensating their hypothetical reduced abundance, and 2) increased bare soil could have r-selected a different species composition, e.g. vagrant lycosid fauna, which appreciate open habitats [73], [90].

Differently from that soil-related feeding strategy, the orb-weaving spiders converged to rosette-form plants and to plant FD (Fig. 3), a surrogate for structural diversity of vegetation. Several works have already demonstrated the importance of habitat architecture to foraging site selection and orb-web spider establishment [93], [94], [95]. Because species and individuals of different ages differ in web size, mesh spacing and web position within the vegetation, the greater architectural heterogeneity, the more web-attachment sites are provided and the higher the density of orb-web spiders supported. Rosettes, and especially *Eryngium horridum* individuals, which present a vertical architectural stratification, seemed to offer suitable web sites. There are some evidences of orb-weaving spiders associations with this life form [96], [97], and the spider benefits might involve stable web support due to leaf arrangement, potential source of moisture and opportunity to catch flying insects taking off from the plant's conical base.

Finally, we showed that the gradient of plant FD driven by fire history in grasslands influenced not only the orb-weavers, but also the functional diversity of the entire spider community, leading to a trait-divergence assembly pattern (Fig. 3) [13]. Although it is well accepted that invertebrate natural enemies densities and diversity are promoted in complex-structured habitats [94], [95], no study to date has shown the close association between FD of vegetation and spiders, especially considering the morphological traits of the later. In our system, spider individuals occurring in more functionally diverse plant communities were more diverse regarding their body, chelicerae and eye sizes and also to their web building type (orb or irregular web) compared to the less functionally diverse plant communities where spiders were more similar to each other concerning the same traits. These findings support the habitat heterogeneity hypothesis [98], [99] at the individual level, demonstrating the coexistence of individuals with segregated functional traits in a more complex vegetation, probably due to niche diversification and increased possibility of resource exploitation (foraging sites, prey, shelters). Further, we would expect such habitat heterogeneity to reduce spider competition for similar resources [14], [19], and to decrease antagonist interactions such as intraguild predation [100] with increasing plant FD, bringing positive effects on prey suppression efficiency [101].

Fire is an important force modelling biodiversity in South Brazilian grasslands, but its effects on spider fauna communities are transient, lasting less than one year, as shown in our small scale experiment that mimics a patchy fire mosaic. This has implications for management, indicating that low intensity, patchy burnings, which would be the case when flammable biomass accumulation is low, would not harm grassland spider communities. Our results strongly suggest that the incorporation of information on the organisms' functional traits into biodiversity monitoring can provide a mechanistic understanding of the response of communities to environmental change, especially when more than one trophic level is considered [5]. Instead of considering the species as a functional unit, individual-based trait data was a realistic and successful way of revealing patterns of spider community assembly after fire, since the entire trait variability of the system was considered [36]. Specifically, we showed patterns indicative of environmental filtering and limiting-similarity processes driven by the fire-induced vegetation change.

## Supporting Information

Table S1 Description of spider data. Family affiliation used in the classification of spider feeding strategies, and number of individuals sampled in soil and vegetation in the study.
(DOCX)

Table S2 Summary of the results of plant functional resilience to fire. Probability values from analysis of variance in blocks with permutation test obtained for plant community indices between control and burned plots before and after fire (a.f). For CWM_all traits the analysis was multivariate. In case of significant differences, positive or negative effects of fire are showed in brackets.
(DOCX)

Figure S1 Matrices used in statistical analyses. Spider matrices are described by B_S_ (individuals by traits), W_S_ (plots by the presence of the individuals), and F_S_ (plots by the abundance of individuals classified in families). Plant matrices are described by B_P_ (species by traits), W_P_ (plots by the cover of species), and the environmental vectors E_PB_ (plots by aboveground biomass) and E_FD_ (plots by plant functional diversity). Matrix T is computed by matrix multiplication T = WB for both spiders (T_S_) and plants (T_P_), and represent community weighted mean traits (CWM).
(TIF)

Figure S2 Spider community resilience to fire. Mean (±SE) of spider individual's abundance (log transformed) (A) and family Simpson diversity (B) in control (light-green symbols) and burned (dark-orange symbols) plots; and similarity coefficients of spider family composition (C) between control and burned plots in different sampling dates (before fire, 1, 6–7, 12 months after fire). Probability values from analysis of variance (A and B in blocks) with permutation tests are presented.
(TIF)
