# Supplementary material for: Spider Trait Assembly Patterns and Resilience under Fire-Induced Vegetation Change in South Brazilian Grasslands
Source: PLoS One. 2013 Mar 28;8(3):e60207. doi: 10.1371/journal.pone.0060207 (PMC3610671; doi:10.1371/journal.pone.0060207)
Supplement: Table S1 — Description of spider data. Family affiliation used in the classification of spider feeding strategies, and number of individuals sampled in soil and vegetation in the study. (DOCX) [file pone.0060207.s001.docx]

| **Feeding strategies** | **Spider families** | **Number of individuals** | | |
| --- | --- | --- | --- | --- |
|  |  | **Soil** | **Vegetation** | **Total** |
| Orb web | Araneidae, Tetragnathidae | 5 | 413 | 418 |
| Irregular web | Amaurobiidae, Dipluriidae, Hahniidae, Linyphiidae, Pholcidae, Pisauridae, Theriididae, Titanoecidae | 141 | 49 | 190 |
| Ground hunters | Corinnidae, Ctenidae, Gnaphosidae, Lycosidae, Miturgidae, Oonopidae, Salticidae | 192 | - | 192 |
| Vegetation hunters | Anyphaenidae, Miturgidae, Oxyopidae, Philodromidae, Salticidae, Sparassidae, Thomisidae | - | 955 | 955 |
| **Total** | | 353 | 1402 | 1755 |
