# Supplementary material for: Spider Trait Assembly Patterns and Resilience under Fire-Induced Vegetation Change in South Brazilian Grasslands
Source: PLoS One. 2013 Mar 28;8(3):e60207. doi: 10.1371/journal.pone.0060207 (PMC3610671; doi:10.1371/journal.pone.0060207)
Supplement: Table S2 — Summary of the results of plant functional resilience to fire. Probability values from analysis of variance in blocks with permutation test obtained for plant community indices between control and burned plots before and after fire (a.f). For CWM_all traits the analysis was multivariate. In case of significant differences, positive or negative effects of fire are showed in brackets. (DOCX) [file pone.0060207.s002.docx]

| **Measures of plant community** | | **p-values (fire effect)** | |
| --- | --- | --- | --- |
|  |  | **Before fire** | **9 month a.f.** |
| FD | All traits | 0.804 | 0.126 |
|  | Morphological | 0.843 | 0.295 |
|  | Life-form | 0.667 | **0.036 (+)** |
| CWM | All traits | 0.642 | **<0.001** |
|  | Plant height | 0.863 | **0.003 (-)** |
|  | Leaf width | 0.902 | **0.009 (+)** |
|  | Leaf length | 0.180 | 0.686 |
|  | Leaf area | 0.706 | 0.190 |
|  | Graminoids | 0.583 | **0.057 (-)** |
|  | Forbs | 0.541 | 0.643 |
|  | Rosette | 1.00 | **0.033 (+)** |
