# Supplementary figures and images for: Spider Trait Assembly Patterns and Resilience under Fire-Induced Vegetation Change in South Brazilian Grasslands
Source: PLoS One. 2013 Mar 28;8(3):e60207. doi: 10.1371/journal.pone.0060207 (PMC3610671; doi:10.1371/journal.pone.0060207)

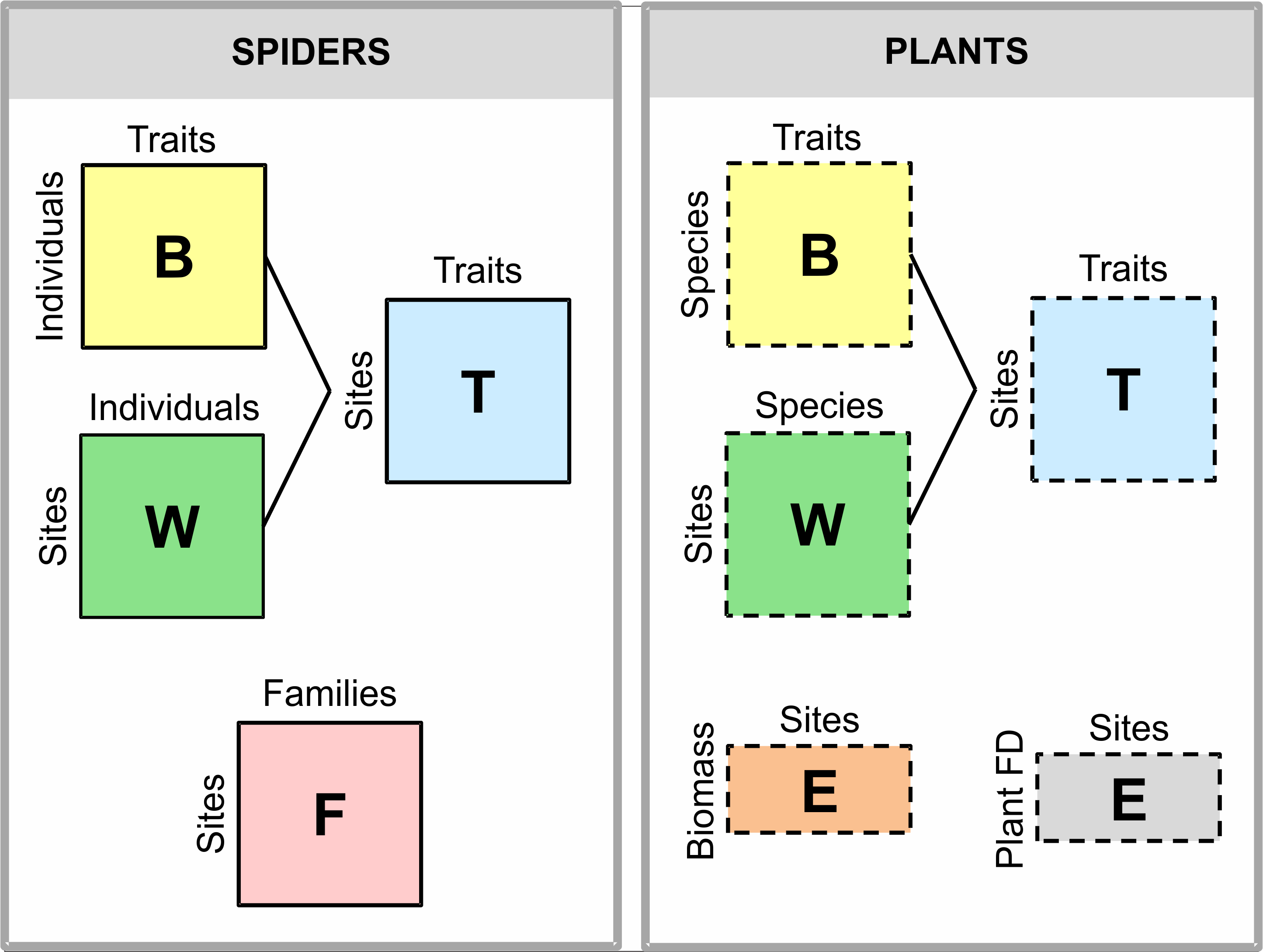

Supplement: Figure S1 — Matrices used in statistical analyses. Spider matrices are described by BS (individuals by traits), WS (plots by the presence of the individuals), and FS (plots by the abundance of individuals classified in families). Plant matrices are described by BP (species by traits), WP (plots by the cover of species), and the environmental vectors EPB (plots by aboveground biomass) and EFD (plots by plant functional diversity). Matrix T is computed by matrix multiplication T = WB for both spiders (TS) and plants (TP), and represent community weighted mean traits (CWM). (TIF) [file pone.0060207.s003.tif]

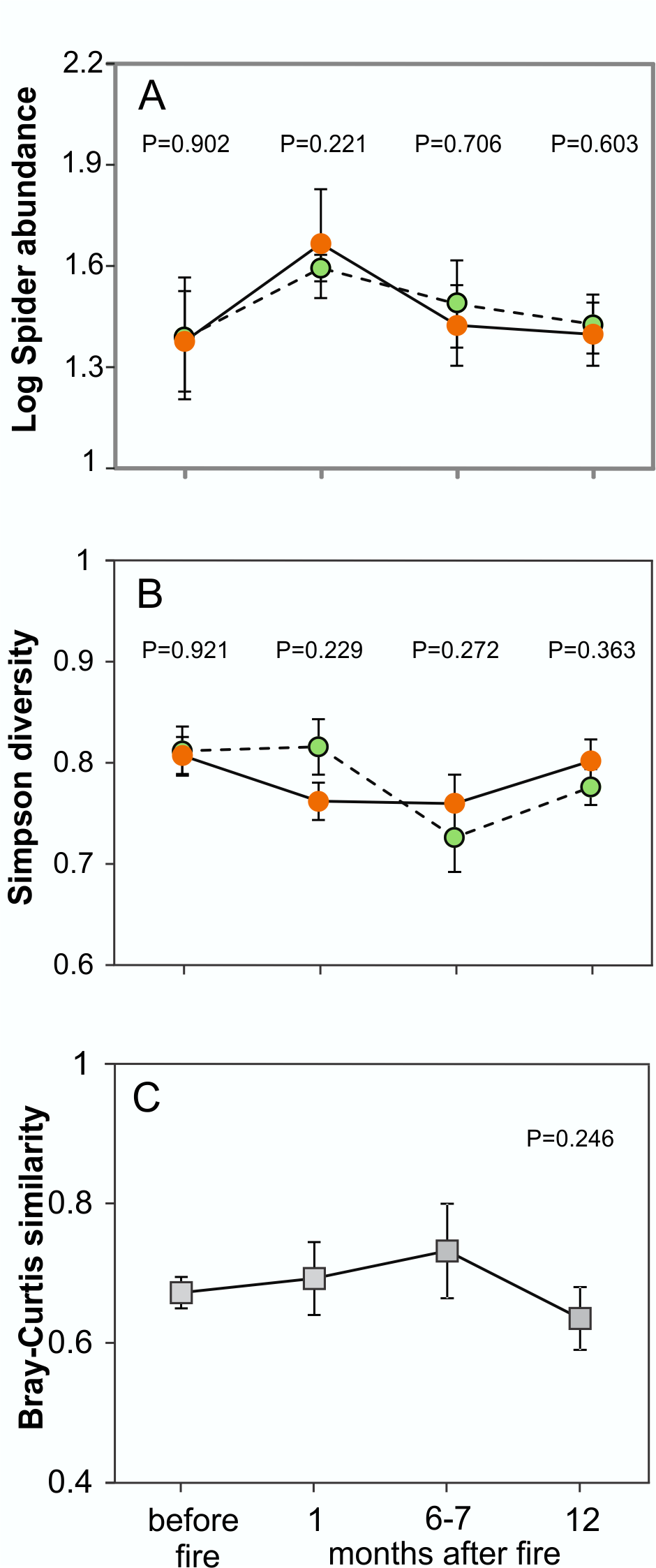

Supplement: Figure S2 — Spider community resilience to fire. Mean (±SE) of spider individual's abundance (log transformed) (A) and family Simpson diversity (B) in control (light-green symbols) and burned (dark-orange symbols) plots; and similarity coefficients of spider family composition (C) between control and burned plots in different sampling dates (before fire, 1, 6–7, 12 months after fire). Probability values from analysis of variance (A and B in blocks) with permutation tests are presented. (TIF) [file pone.0060207.s004.tif]
